# Designs for Vaccine Studies

**DOI:** 10.1146/annurev-statistics-033121-120121

**Published:** 2024-10-30

**Authors:** M. Elizabeth Halloran

**Affiliations:** 1 Vaccine and Infectious Disease Division, Fred Hutchinson Cancer Center, Seattle, Washington, USA;; 2 Department of Biostatistics, University of Washington, Seattle, Washington, USA

**Keywords:** causal inference, cluster-randomized trial, immune correlates, observational study, regression discontinuity design, ring vaccination, target trial emulation, test-negative design, vaccines

## Abstract

Due to dependent happenings, vaccines can have different effects in populations. In addition to direct protective effects in the vaccinated, vaccination in a population can have indirect effects in the unvaccinated individuals. Vaccination can also reduce person-to-person transmission to vaccinated individuals or from vaccinated individuals compared with unvaccinated individuals. Design of vaccine studies has a history extending back over a century. Emerging infectious diseases, such as the SARS-CoV-2 pandemic and the Ebola outbreak in West Africa, have stimulated new interest in vaccine studies. We focus on some recent developments, such as target trial emulation, test-negative design, and regression discontinuity design. Methods for evaluating durability of vaccine effects were developed in the context of both blinded and unblinded placebo crossover studies. The case-ascertained design is used to assess the transmission effects of vaccines. The novel ring vaccination trial design was first used in the Ebola outbreak in West Africa.

## 1. INTRODUCTION

Vaccine efficacy and vaccine effectiveness (VE) are generally estimated as one minus some measure of relative risk, RR, in the vaccinated group compared with the unvaccinated group:

1. VE=1-RR.

The groups being compared could be composed of individuals or of populations or communities. Vaccine efficacy and effectiveness is often given in percent, so the fraction is multiplied by 100.

For example, in the primary placebo-controlled, observer-blinded efficacy trial of the BNT162b2 mRNA COVID-19 vaccine, one vaccine efficacy estimate was in participants from 7 days after the second dose to the end of the surveillance period, if they had no evidence of infection before that period (Polack et al. 2020). In the vaccine group, there were 8 cases in 2.214 1,000 person-years surveillance time, and in the placebo group, there were 162 cases in 2.222 1,000 person-years surveillance time. Vaccine efficacy (%) was estimated to be 1 –(8/2.214)/(162/2.222) as 95.0% [95% confidence interval (CI), 90.0%−97.9%].

Design of vaccine studies has a history extending back over a century. Particularly since the SARS-CoV-2 pandemic and the widespread use of COVID-19 vaccines, new studies to evaluate vaccines have been conducted. It has become widely known that individually randomized, double-masked, controlled trials with confirmed, symptomatic disease as outcome do not tell the whole story. Asymptomatic infection, infectiousness, transmission, and indirect effects of vaccination are important. Real-world evidence of how vaccines work in settings other than controlled vaccine trials is needed. Halloran et al. (2010) give a wide-ranging overview in their book *Design and Analysis of Vaccine Studies*. They cover the history of vaccine trial design and details about how to assess the different effects of vaccination. In this article, we review some of the concepts covered there, but we focus on several new developments in vaccine study design that were not contained in their book.

## 2. OVERVIEW OF VACCINE EFFECTS

### 2.1. Hierarchy of Vaccine Parameters of Direct Effects

In 1916, Sir Ronald Ross published his treatise on the Theory of Happenings. Happenings can be divided into two classes, namely those in which the frequency of the happening is independent of the number of individuals already affected, and those in which the frequency of the happening depends on this quantity. Due to dependent happenings in infectious disease, vaccination can produce several different kinds of effects at the individual and the population level. In an individual, vaccination can induce a biologically protective response against infection and/or disease and/or reduce the degree or duration of infectiousness for other individuals. Widespread vaccination in a population can reduce transmission and produce indirect effects, even in individuals who were not vaccinated.

Halloran et al. (1997) presented a systematic framework showing the relation among many of the different vaccination effects and the parameters and study designs to estimate them. One is the vaccine efficacy for susceptibility, $VE_{\text{S}}$, which is the efficacy of a vaccine to reduce the risk of infection. The measure of risk can be a form of the transmission probability, which conditions on exposure to infection, or the incidence rate, hazard rate, or cumulative incidence (attack rate), which do not condition on exposure to infection.

Rhodes et al. (1996) used counting process models to demonstrate that these commonly used relative risk parameters form a hierarchy requiring different amounts of information about the contact and infection processes. Heuristically, because of the dependent happening structure of events in infectious diseases, there is an intrinsic relation among the different parameters on which the $VE_{\text{S}}$ estimators are based. Let p denote the transmission probability, the probability of transmission during a contact of a susceptible person with an infective person. Let c(t) be the contacts per time and P(t) be the prevalence of infective individuals in the population at time t. Let the hazard rate λ(t) be the event rate at time t; similarly, the incidence rate is the events per person-time. The hazard rate can be expressed as

2. λ(t)=c(t)pP(t).

Expression 2 represents the fundamental dependent happening relation of Ross (1916), where the transmission probability has a central role. Even if the different components of the hazard rate are not measured, we can consider the underlying process that is producing the infections that we observe. Similarly, the cumulative incidence CI(t) at some time t is a function of the hazard rate during the follow-up period, and thus also a function of the contact rate, the transmission probability, and the prevalence of infection in the contacts,

3. $$CI(t)=1-\text{exp}\left[-\int_{0}^{t}\lambda (u)\text{d}u\right]=1-\text{exp}\left[-\int_{0}^{t}c(u)pP(u)\text{d}u\right].$$

Even though the cumulative incidence is something of a black-box estimator, it is useful to think about the underlying transmission system that would produce the observed events.

Often a vaccine trial outcome is based on disease, not infection. Then vaccine efficacy may be denoted $VE_{\text{SP}}$ to denote infection with progression to disease. Most vaccine trials do not use different notation in their reports.

### 2.2. Effects of Vaccines on Person-to-Person Transmission

As mentioned above, the direct protective effect of vaccination can be estimated from the relative transmission probability in vaccinated compared with unvaccinated individuals who are exposed to infection. The efficacy of a vaccine in reducing infectiousness, $VE_{\text{I}}$, can be estimated epidemiologically by comparing the per-contact transmission probability from vaccinated people who become infected with the transmission probability from unvaccinated people who become infected. The relative risk comparison groups are defined according to the vaccination status of the infectious person contacting the susceptible person. There are at least seven measures potentially of interest in estimating the effect of vaccines on transmission, depending on whether one stratifies on the vaccination status of the exposing individual, or the exposed individual, or both.

### 2.3. Indirect, Total, and Overall Effects

Halloran & Struchiner (1991) define study designs for dependent happenings that allow evaluation of the indirect, total, and overall effects of vaccination. The population effects of vaccination are defined within the context of a certain vaccination program, or allocation of vaccination, and thus the unit of inference is the population. Several populations or communities need to be included in the study to take potential variability into account. Suppose there are two populations, A and B, and that no vaccination has taken place in population B, and a proportion of the people are vaccinated in population A. The indirect effect of the vaccination strategy is defined by the comparison of the incidence or outcome of interest in the unvaccinated people in population A compared with that in unvaccinated people in population B. The total effect of the combination of being vaccinated and the strategy is the outcome in the vaccinated people in the population A compared with that of the unvaccinated people in population B. The overall effectiveness of the vaccine and allocation strategy compares the average outcomes in population A with the average outcomes in population B.

## 3. RANDOMIZED TRIALS

### 3.1. Individually Randomized Trials

The individually randomized, double-masked, controlled trial remains the gold standard for Phase III primary efficacy and safety studies of vaccines. These are well-described elsewhere. Here we focus on a couple of recent developments. Evaluation of safety is important in all vaccine trials but is outside the scope of this article.

### 3.2. Durability and Waning

As a randomized trial continues, a key issue is the possibility that vaccine efficacy may wane over time. If efficacy is demonstrated, the treatment of placebo volunteers becomes an issue. Were all participants in the trials to continue on their randomized assignment (study vaccine or placebo), evaluation of potential waning of vaccine efficacy would be straightforward. For COVID-19 vaccine trials, there was broad consensus that placebo volunteers should be offered a vaccine once efficacy had been established. Crossover of placebo participants to vaccine complicates inference about long-term durability of vaccine efficacy.

#### 3.2.1. Placebo crossover trials with blinding intact.

Fintzi & Follmann (2021) show how to analyze durability following placebo crossover. They demonstrate that the vaccine efficacy profile that would be observed in a placebo controlled trial is recoverable in a trial with placebo crossover. Their approach assumes that the participants are still blinded to their assignment. When the placebo recipients are vaccinated, the individuals who were originally vaccinated receive a placebo. Thus, the trial remains randomized and blinded, even though there is no longer an unvaccinated control group. The trial is divided into two time periods: The first is before the crossover, and the second is after the crossover. The general idea is that in the second period of the trial, one observes the number of cases in the newly vaccinated crossover arm. From the efficacy estimated in the precrossover interval of the trial, one estimates how many cases one would have expected in the placebo arm in the second period had they not crossed over. By contrasting this counterfactual placebo case count to the number of cases observed in the original vaccine arm during the second period, one can estimate the vaccine efficacy in the second period. By comparing this to the estimate of vaccine efficacy in the first period, one can conclude whether vaccine efficacy has waned or not. The result holds no matter when the crossover occurs and with no assumptions about the form of the efficacy profile. The method requires only that the vaccine efficacy profile applies to the newly vaccinated participants irrespective of the timing of vaccination. Confounding due to unblinding is not addressed because this approach assumes that participants remain blinded. Fintzi & Follmann (2021) present the technical development of the result and simulated trials.

#### 3.2.2. Placebo crossover trials with unblinding.

Tsiatis & Davidian (2022) propose a statistical framework based on data where the participants are unblinded and those on placebo may choose to cross over to study vaccine. In their setting, ethical considerations dictate that trial participants be unblinded and those randomized to placebo be offered study vaccine, leading to trial protocol amendments specifying unblinding strategies. Those who were originally in the vaccine arm are also unblinded and continue under observation. Tsiatis & Davidian (2022) focus on the particular features of the Moderna COVID-19 mRNA vaccine trial. They propose a statistical framework based on a potential outcomes formulation within which they develop methods for inference on potential waning of vaccine efficacy over time and estimation of vaccine efficacy at any postvaccination time. They address the issue that the placebo recipients who choose to get the vaccine may be more or less likely to be infected. They develop their conceptual framework from the dependent happenings viewpoint as in Expression 2. The probability that a trial participant will become infected depends on the population outside the clinical trial, including the contact rate c(t), the transmission probability p(t), and the prevalence of infections P(t) at time t. The COVID-19 vaccine trials took place in difference sites, so prevalence was allowed to vary by time and site. The population at large is considered unaffected by the clinical trial participants. A similar framework is developed for the individual trial participants that allows for waning of vaccine efficacy. Five identifiability assumptions are presented. Under these five assumptions, unbiased estimating equations based on the observed data are developed that yield consistent and asymptotically normal estimators for the parameters for waning. They use inverse probability weighting to probabilistically represent potential outcomes in terms of the observed data to mimic the estimating equations. Because of the unblinding, confounding is addressed through a potential outcomes formulation and inverse probability weighting.

#### 3.2.3. Another approach without a crossover trial.

Another approach for testing for and estimating time-varying vaccine efficacy not in the context of crossover is using smoothed scaled Schoenfeld residuals (Schoenfeld 1982) from a Cox proportional hazards model. Fong et al. (2018) reanalyzed a cluster-randomized trial of a bivalent killed whole-cell cholera vaccine in Kolkata, India. Efficacy did not appear to wane in adult vaccinees, but there were insufficient data to assess waning among child vaccinees. Rane et al. (2021) used this approach to assess durability of protection after five doses of acellular pertussis vaccine among 5–9-year-old children in King County, Washington. They did not find evidence of waning of vaccine effectiveness for up to 4 years after five doses of acellular pertussis vaccine among these children. Rane et al. (2021) report the software used to do the analysis.

### 3.3. Vaccine Trials for Emerging Infectious Diseases

Public health emergencies, such as an Ebola disease outbreak, provide a complex and challenging environment for the evaluation of candidate vaccines. In response to the Ebola outbreak in West Africa, the World Health Organization (WHO) created the WHO Research and Development Blueprint to prepare for various aspects of emerging infectious disease threats.

#### 3.3.1. Ring vaccination trial design.

The Ebola outbreak in West Africa in 2014–2016 focused attention on the need for more flexible vaccine trial designs for emerging infectious diseases. During that outbreak, by the time the vaccine trials were ready to go to the field, transmission was already waning. The Ebola ring vaccination trial (Ebola ça Suffit Ring Vaccination Trial Consortium 2015) was proposed to take the vaccine trial where the transmission was occurring. After confirmation of a case of Ebola virus disease, the design was to enumerate on a list a ring (cluster) of all of their contacts and contacts of contacts, including named contacts and contacts of contacts who were absent at the time of the trial team visit. Rings are randomized 1:1 to (*a*) immediate vaccination of eligible adults with single dose vaccination or (*b*) vaccination delayed by 21 days. Vaccine efficacy against disease is assessed in participants over equivalent periods from the day of randomization. Ring vaccination trials are adaptive, can be run until disease elimination, allow interim analysis, and can go dormant during interepidemic periods.

Henao-Restrepo et al. (2017) report the final results of the Ebola ring vaccination trial using the randomization of immediate versus delayed vaccination of the rings. It was an open-label, cluster-randomized ring vaccination trial in the communities of Conakry and eight surrounding prefectures in the Basse-Guinée region of Guinea, and in Tonkolili and Bombali in Sierra Leone. They assessed the efficacy of a single dose of rVSV-ZEBOV (which stands for recombinant vesicular stomatitis virus-Zaire Ebola virus) in the prevention of laboratory-confirmed Ebola virus disease. No cases of Ebola virus disease occurred 10 days or more after randomization among randomly assigned contacts and contacts of contacts vaccinated in immediate clusters versus 16 cases (7 clusters affected) among all eligible individuals in delayed clusters. Estimated vaccine efficacy was 100% (95% CI, 68.9%−100.0%).

#### 3.3.2. Platform trials and core protocols.

A committee on clinical trial design was part of the WHO Blueprint. Dean et al. (2019) outline the need for flexible and responsive vaccine trial designs to be used in public health emergencies. Because outbreaks of emerging infectious diseases can die out before the number of events needed for the power of the study is achieved, concern about publication bias led to an emphasis on the need to report the results of all clinical trials, even those with inconclusive results at the end of an outbreak (Dean et al. 2020a). The WHO committee on clinical trial design proposed the use of core protocols (also called master protocols). The core protocol concept would allow a clinical trial to extend across multiple infectious disease outbreaks. The approach would be useful in the face of the unpredictable features of an epidemic.

Building on knowledge and experiences in the designs of vaccine efficacy trials against other pathogens, Longini et al. (2022) develop designs of randomized Phase III vaccine efficacy trials for Marburg virus vaccines. A core protocol approach, also called a platform trial, would be used, allowing multiple vaccine candidates to be tested against controls. The primary objective of the trial would be to evaluate the effect of each vaccine on the rate of virologically confirmed Marburg virus disease, although Marburg infection assessed via seroconversion could be the primary objective in some cases. The overall trial design could be a mixture of individually randomized and cluster-randomized designs, with individual randomization done whenever possible. Clusters would consist of either contacts and contacts of contacts of index cases, that is, ring vaccination, or other transmission units. A vaccine would be considered successful if its estimated efficacy is greater than 50% and has sufficient precision to rule out that true efficacy is less than 30%. This would require approximately 150 total endpoints, that is, cases of confirmed Marburg virus disease, per vaccine/comparator combination. The proposed trial design would be applicable to other pathogens against which effective vaccines are not yet available.

## 4. OBSERVATIONAL STUDIES

After a vaccine has been licensed or at least approved for emergency use, it is often unethical to conduct placebo-controlled randomized studies. When a randomized study cannot be conducted because it is either unfeasible or unethical, then observational data may be used.

### 4.1. Emulating Target Trials with Big Data

Hernan & Robins (2016) propose that causal inference from large observational databases could be viewed as an attempt to emulate a randomized experiment. This would be the target experiment or target trial that would answer the question of interest.

#### 4.1.1. Target trial emulation.

Hernan & Robins (2016) outline a framework for effectiveness research using big data that makes the target trial explicit when the randomized trial is not available. One begins by outlining the target trial protocol. For vaccination, this includes the eligibility criteria, vaccination strategies, assignment procedures, follow-up period, outcome (say infection or laboratory-confirmed symptomatic infection), causal contrasts of interest (intention-to-treat or per-protocol effect), and analysis plan. Defining the baseline, or time zero, for beginning follow-up in the observational data is necessary when emulating a target trial.

Randomized clinical trials are considered the gold standard for evaluating vaccines, but they also have limitations (Dagan et al. 2021). These include sample size and subgroup analysis, restrictive inclusion criteria, and a highly controlled setting that may not be replicated in a mass vaccine rollout. Thus, making use of large databases using target trial emulation has some advantages over randomized trials. With international attention on the effectiveness of COVID-19 vaccination, real-world evidence increased in importance as the various vaccines were rolled out in different settings. Emulating target trials using large administrative databases and electronic health records was possible in some contexts. Because of the growing importance of the use of target trial emulation to assess vaccine effectiveness, we present an example of such a study, whereby the reader is referred to the original paper and supplement for more detail.

#### 4.1.2. Target trial emulation for COVID-19 vaccine effectiveness.

The target trial emulation paradigm guided the evaluation of mass vaccination with COVID-19 vaccines across diverse populations in uncontrolled settings. One of the first was the study of vaccination from December 20, 2020, to February 1, 2021, with the Pfizer mRNA vaccine in Israel (Dagan et al. 2021). The study analyzed data from the Clalit Health Services, the largest of four integrated health services organizations in Israel, which insures 4.7 million people (53% of the population). The investigators explicitly designed the observational study to emulate a target trial of the causal effect of the Pfizer mRNA vaccine on COVID-19 outcomes. Eligibility criteria were defined. These included being age 16 years or older, not having a previously documented positive SARS-CoV-2 polymerase chain reaction (PCR) test, and being a member of the health care organization during the previous 12 months. Exclusion criteria were also defined.

Each day during the study period, all newly vaccinated persons were matched in a 1:1 ratio to unvaccinated controls. For each person, follow-up ended with the earliest occurrence of the outcome event, death unrelated to COVID-19, vaccination (for unvaccinated controls), vaccination of the matched control (for vaccinated persons), or the end of the study period. Vaccine recipients and controls were matched on variables associated with probability of both vaccination and infection or severity of COVID-19, history of influenza vaccination during the preceding 5 years, and pregnancy, among others. The five outcomes of interest were documented SARS-CoV-2 infection confirmed by positive PCR test, documented symptomatic COVID-19, hospital admission for COVID-19, severe COVID-19, and death from COVID-19. They also evaluated an additional outcome, SARS-CoV-2 infection without symptoms, as an imperfect proxy for asymptomatic infections (because mild symptoms may not be documented).

Follow-up was divided into three periods, days 14 through 20 after the first dose of vaccine, days 21 through 27 after the first dose (administration of the second dose was scheduled for day 21 after the first dose), and day 7 after the second dose until the end of the follow-up. In the Clalit Health Services, 1,163,534 vaccinated members were eligible for the study, of whom 596,618 were matched to unvaccinated controls. During a mean follow-up of 15 days, 10,561 infections were documented. The cumulative incidence curves of the vaccinated and unvaccinated members were mostly overlapping in the first 10 to 14 days of follow-up, indicating a good match of the two groups.

Survival curves for the vaccinated and unvaccinated groups were estimated with the Kaplan-Meier estimator. For each of the three periods, they used the Kaplan-Meier estimator with daily outcome and censoring events to compute the probability of the outcome during the period, using matched pairs in which both persons were still at risk at the beginning of the period. Then the risk ratios for vaccination as compared with no vaccination were calculated. Vaccine effectiveness was estimated as one minus the risk ratio, as in Equation 1.

During the 14 to 20 days after the first dose, estimated vaccine effectiveness for documented infection was 46% (95% CI, 40%−51%) and for severe illness was 62% (95% CI, 39%−80%). During the period from 21 to 27 days after the first dose, estimated effectiveness for these outcomes was 60% (95% CI, 53%−66%) and 80% (95% CI, 59%−94%). In the follow-up period starting 7 days after the second dose, vaccine effectiveness for documented infection and severe illness was 92% (95% CI, 88%−95%) and 92% (95% CI, 75%−100%). Vaccine effectiveness estimates were also provided in subpopulations according to baseline covariates, such as gender, age, and coexisting conditions.

The Dagan et al. (2021) study illustrates the power of using big data to evaluate vaccine effectiveness by emulating a target trial. This study had many more severe disease outcomes than the initial randomized study of this vaccine. The large sample size in this study also allowed estimation of vaccine effectiveness in specific subpopulations that the randomized trial was not sufficiently powered to evaluate.

#### 4.1.3. Target trial emulation for comparing effectiveness of interdose intervals.

Shioda et al. (2024) used a target trial emulation approach to compare the effectiveness of different interdose intervals among >6 million mRNA vaccine recipients in Georgia, USA, from December 2020 to March 2022. They used a clone-censor weight analysis in the target trial emulation. This method mimics a per-protocol randomized controlled trial in which individuals are randomly allocated to different dosing protocols. They compared three protocols defined by interdose interval: recommended by the US Food and Drug Administration (FDA) (17–25 days for Pfizer-BioNTech, 24–32 days for Moderna), late-but-allowable (26–42 days for Pfizer-BioNTech, 33–49 days for Moderna), and late (≥43 days for Pfizer-BioNTech, ≥50 days for Moderna). The clone-censor weight method creates three copies of the longitudinal dataset corresponding to the three dosing schedules being considered. In the short term, the risk of SARS-CoV-2 infection was lowest under the FDA-recommended protocol. Longer-term, the late-but-allowable protocol resulted in the lowest risk [risk ratio on Day 120 after the first dose administration compared with the FDA-recommended protocol: 0.83 (95% CI, 0.82–0.84)]. Using the target trial emulation with clone-censor weights, they showed that delaying the second dose by 1–2 weeks may provide stronger long-term protection.

### 4.2. Test-Negative Design

The test-negative design is a fairly recent development for evaluating vaccines (Jackson & Nelson 2013). In the test-negative design, routine testing at health care facilities is leveraged to estimate the effectiveness of a vaccine. Individuals presenting at health care facilities with a particular syndrome that has the symptoms of the disease of interest targeted by the vaccine being evaluated are enrolled in the study. They are then tested for the pathogen of interest. Those individuals who test positive for the pathogen of interest are the cases, and those who test negative are the noncases. For example, under the test-negative design to evaluate influenza vaccine effectiveness, study participants are all people who seek care for an acute respiratory illness with certain symptoms. Vaccine effectiveness is estimated from the ratio of the odds of vaccination among the participants who test positive for influenza to the odds of vaccination in those who test negative (odds ratio OR), adjusting for key confounders:

4. VE=1-OR.

A key assumption is that the vaccine of interest has no effect on the other causes of the same clinical syndrome.

The test-negative design has some advantages. Because the people are coming to the clinic and being tested in any event, it is an inexpensive design. The study is not a classical case-control design, because it does not draw the controls from a population, but that also makes it less expensive. The vaccination status must still be ascertained. Some researchers believe that the test-negative design is less susceptible to bias from confounding due to health-seeking behavior relative to traditional case-control or cohort studies. Sullivan et al. (2016) examined the theoretical basis of the test-negative design for influenza vaccine effectiveness. Westreich & Hudgens (2016) raised doubts about the approach. Dean et al. (2020b) examined temporal confounding by generalizing derivations to allow for time-varying vaccine status, including out-of-season controls, and open populations. They confirm that calendar time is an important confounder when vaccine status varies during the study. They demonstrate that, when time is not a confounder, including out-of-season controls can improve precision.

De Serres et al. (2013) compared the vaccine effectiveness estimates from a test-negative design to those from randomized placebo-controlled trials by assuming that the test-negative design study had been conducted in the trial population. The efficacy estimates and their CIs were virtually identical for the per-protocol randomized controlled trial versus test-negative design analyses of influenza vaccine. Schwartz et al. (2017) compared the estimates for randomized trials of two rotavirus vaccines in low-income settings with the estimates had test-negative designed studies been conducted in the trial populations. The estimates based on the two approaches were very similar. Mésidor et al. (2024) provided a systematic methodology review of test-negative design studies to estimate effectiveness of COVID-19 vaccination.

Feldstein et al. (2021) explore advantages and disadvantages of modifying the test-negative design for estimating vaccine effectiveness by using real-time clinically available viral testing results paired with acute respiratory infection eligibility criteria for identifying influenza cases and test-negative controls prior to enrollment. They call this modification the real-time test-negative design (rtTND). They show that the rtTND has the potential to improve influenza vaccine effectiveness studies by optimizing the case-to-test-negative control ratio. It can also have more accurate classification of influenza status, improving study efficiency, reducing study cost, and increasing study power. To limit biases, it is, however, important to have comprehensive clinical influenza testing at study sites and accurate influenza tests.

### 4.3. Regression Discontinuity Design

The regression discontinuity design is a quasi-experimental design first proposed in educational psychology (Thistlethwaite & Campbell 1960). Basta & Halloran (2019) proposed using this design to evaluate vaccines when eligibility for vaccination is based on a defined cutoff such as age or grade in school. It assumes that, in a small neighborhood around the cutoff, treatment assignment is ignorable, so that locally the potential outcome is independent of assignment, as in a randomized study. Thus, the regression discontinuity design can be used to estimate the local treatment effect around a small neighborhood at the cutoff that defines eligibility for the treatment. The approach is particularly well-suited to evaluating vaccines when a vaccination program is designed to target a specific group defined by a continuous variable with a clear cutoff that establishes eligibility for the vaccine. The regression discontinuity design could also be used to evaluate vaccines when incidence of disease is very low, particularly if the disease of interest can be assessed using routinely collected surveillance data.

## 5. VACCINE EFFECTS ON TRANSMISSION

Studies to assess vaccine effects on transmission generally need to take place in transmission units. The idea of a transmission unit is that individuals make contact sufficient for transmission within it. A common choice of transmission unit is the household. Historically, household studies focused on evaluating the protective effects of vaccination on the exposed, $VE_{\text{S}}$. In recent years, interest has grown in the vaccine effect on the ability to transmit the infection from vaccinated infected people compared with unvaccinated infected people, $VE_{\text{I}}$. The analysis is often based on the relative secondary attack rate (SAR) between the infected individuals of interest. The SAR is a special case of the transmission probability. The SAR is the probability that an individual infects another person during some period of time.

### 5.1. Household Studies Using Population Surveillance Data

Préziosi & Halloran (2003) evaluated vaccine effects on transmission of symptomatic pertussis in Niakhar, Senegal. The design of this study leveraged the active population surveillance that had taken place since 1983 in Niakhar, a rural community of 30 villages. A randomized trial of two pertussis vaccines in young children had been conducted, but some individuals did not get vaccinated. Extended families were under longitudinal surveillance. All households (compounds) were visited weekly. In addition to the usual demographic variables, pertussis vaccination status and vaccination dates were known. Using a wide spectrum of case definitions, vaccine efficacy was estimated as 1 – the ratio of SAR in all households with cases during the calendar year 1993, a pertussis epidemic year. In particular, vaccine efficacy for infectiousness, $VE_{\text{I}\cdot }$, was estimated comparing the SAR from vaccinated infected cases, $SAR_{1\cdot }$, to the SAR from unvaccinated infected cases, $SAR_{0\cdot }$. The · indicates here that the exposed individuals are not being stratified by vaccine status:

5. $$VE_{\text{I}\cdot }=1-\frac{SAR_{1\cdot }}{SAR_{0\cdot }}.$$

Inference was based on using bootstrap by household (compound) to take within-household correlation into account. Estimated vaccine efficacy for infectiousness was 85% (95% CI, 46%−95%) for children vaccinated with three doses of a whole-cell (94%) or an acellular (6%) pertussis vaccine, with pertussis defined as a cough ≥21 days with paroxysms confirmed by culture, serology, or contact with a culture-confirmed person. It was high for all case definitions. Partial vaccination reduced infectiousness. The conclusion was that pertussis vaccination is highly effective in reducing transmission from vaccinated breakthrough cases, which has been controversial for decades.

### 5.2. Case-Ascertained Household Studies

Rather than having active surveillance of an entire community, a study of the effects of vaccination on transmission can be based on the case-ascertained design. In a case-ascertained design study, households with an individual who tests positive are enrolled if they meet certain eligibility criteria, including being within a certain time of the index case’s illness onset. Recent studies of household transmission of COVID-19, influenza, and other respiratory viruses were conducted using a case-ascertained design to assess whether vaccination reduced the risk of infection within households (Rolfes et al. 2024). Intense follow-up of the contacts used daily symptom diaries, nasal swabbing with viral sequencing, and blood draws. Then prior infection, vaccination status, and hybrid immunity (both vaccination and prior infection) on SARS-CoV-2 infection were assessed by robust, clustered multivariable Poisson regression. The results showed that immunity from COVID-19 vaccination and prior infection was synergistic in protecting household contacts from SARS-CoV-2 infection. A further analysis of the same study could be done looking at how prior infection and vaccination reduce the infectiousness to household contacts. Rolfes et al. (2023) used a similar case-ascertained household study to compare influenza transmission before the COVID-19 pandemic to that in 2021–2022.

## 6. INDIRECT, TOTAL, AND OVERALL EFFECTS

### 6.1. Two-Stage Randomized Trials

Randomized community trials fall into the category of cluster-randomized trials where whole social units, rather than individuals, are randomly assigned to treatment groups. Because vaccines are administered to individuals, randomization can occur at two stages, namely the group level and the individual level within groups. In general an assignment mechanism other than randomization could be in place at the two levels.

Defining causal estimands for indirect, total, and overall vaccine effects is not straightforward. In causal inference, generally the assumption is made that the outcome in one individual is independent of the treatment assignment in other individuals in the study population. This is called the assumption of no interference (Cox 1958). In the dependent happenings in infectious diseases, the assumption of no interference usually does not hold and is the source of the indirect, total, and overall effects of interest. Hudgens & Halloran (2008) defined causal estimands of direct, indirect, total, and overall effects in the presence of interference by positing a population of groups, blocks, or clusters within which interference can occur within the groups but not between groups. This is called the assumption of partial interference (Sobel 2006). Progress has been made in relaxing the assumption of partial interference in the context of networks, but this has been less applied to vaccine studies. We do not consider relaxation of the partial interference assumption further here. The following presentation assumes partial interference. To define the indirect, total, and overall effects of one vaccination strategy compared with another, one needs to consider a second strategy. Hudgens & Halloran (2008) average individual, group, and population outcomes over all possible treatment assignments for a particular allocation strategy or strategies of interest within and across groups. They define causal estimands of the direct, indirect, total, and overall effects that are also averages within the groups and across the population of groups. If randomization is at two stages, that is, randomization of groups to allocation strategies, followed by randomization of individuals within groups according to the vaccination strategy assigned to that group, Hudgens & Halloran (2008) showed that the causal direct, indirect, total, and overall effects are estimable from the observed data. We know of no two-stage randomized vaccine trials.

### 6.2. Other Vaccine Studies for Indirect, Total, and Overall Effects

It could be that vaccination strategies are not randomized at the group level or that vaccination is not randomized at the individual level, or that there is no randomization at either level. In the absence of randomization, Tchetgen Tchetgen & VanderWeele (2012) proposed inverse probability weighted (IPW) estimators of the direct, indirect, total, and overall causal effects in the presence of partial interference based on group-level propensity scores. The estimators entail estimating mean potential outcomes by taking weighted averages of the observed responses where the weights include the inverse of group-level propensity scores. These IPW estimators can be viewed as a generalization of the usual IPW estimator of the causal effect of a treatment in the absence of interference.

To define a group-level propensity score, we present some notation, following Perez-Heydrich et al. (2014). Consider a finite population of N individuals. Suppose the individuals can be partitioned into m groups with $n_{i}$ individuals in group i for i=1,…,m, such that $\sum_{i=1}^{m}n_{i}=N$. Suppose individuals are either vaccinated (a=1) or not vaccinated (a=0). Let $A_{ij}=1$ if individual j in group i is vaccinated, and 0 otherwise. Let ${\text{A}}_{i}=\left(A_{i1},\ldots ,A_{in_{i}}\right)$ denote the vector of vaccination indicators of all individuals in group i. Let ${\text{A}}_{i,-j}=\left(A_{i1},\ldots ,A_{ij-1},A_{ij+1},\ldots ,A_{in_{i}}\right)$ denote the vector of vaccination indicators for all individuals in group i except individual j. Let ${\text{a}}_{i}$ and ${\text{a}}_{i,-j}$ denote realizations of ${\text{A}}_{i}$ and ${\text{A}}_{i,-j}$. Let 𝒜(n) be the set of $2^{n}$ possible vaccination vectors for a group of size n, such that ${\text{a}}_{i}\in 𝒜\left(n_{i}\right)$.

We are assuming partial interference. Let $y_{ij}\left({\text{a}}_{i}\right)$ denote the potential disease outcome (1 if disease or infection, 0 otherwise) for individual j in group i if ${\text{A}}_{i}={\text{a}}_{i}$, such that each individual in group i has $2^{n_{i}}$ potential outcomes. Let $Y_{ij}=y_{ij}\left({\text{A}}_{i}\right)$ denote the observed infection outcome for individual j in group i, and let ${\text{Y}}_{i}=\left(Y_{i1},\ldots ,Y_{in_{i}}\right)$ denote the vector of observed outcomes for all individuals in group i.

The IPW estimators are constructed by weighting the observed individual responses $Y_{ij}$ by the inverse of the group-level propensity score, i.e., the probability a group of individuals receives a particular vaccination vector. When this group-level propensity score is known, Tchetgen Tchetgen & VanderWeele (2012) proved the IPW estimators are unbiased under two assumptions: conditional independence and positivity. Under the conditional independence assumption,

6. $$\text{Pr}\left({\text{A}}_{i}={\text{a}}_{i}|{\text{X}}_{i},{\text{y}}_{i}\right)=\text{Pr}\left({\text{A}}_{i}={\text{a}}_{i}|{\text{X}}_{i}\right),$$

where ${\text{y}}_{i}$ denotes the potential outcomes for all individuals in group i, and ${\text{X}}_{i}=\left({\text{X}}_{i1},\ldots ,{\text{X}}_{in_{i}}\right)$ is an $n_{i}\times p$ matrix with ${\text{X}}_{ij}\;\text{a}\;1\times p$ vector of covariates for individual j in group i. The right side of Equation 6 is the group-level propensity score used in constructing the IPW estimators. Under the positivity assumption,

7. $$\text{Pr}\left({\text{A}}_{i}={\text{a}}_{i}|{\text{X}}_{i}\right)>0,\forall {\text{a}}_{i}\in 𝒜\left(n_{i}\right).$$

Assumptions 6 and 7 are group-level generalizations of the usual no unmeasured confounders and positivity assumptions made at the individual level in the analysis of observational studies when interference is not present.

Perez-Heydrich et al. (2014) used the IPW estimators based on the group-level propensity scores (Equation 6) to estimate the different effects of cholera vaccination based on estimated propensity scores in the presence of interference. In Matlab, Bangladesh, in a study from 1985 to 1988, all children (2–15 years old) and women (>15 years old) were randomly assigned with equal probability to either of two killed cholera vaccines or a placebo. Though this was an individually randomized study, clusters were formed for the estimation of the direct, indirect, total, and overall effects, so that the allocation strategy was not randomized by cluster. Unvaccinated individuals included eligible nonparticipants and placebo recipients. Vaccinated individuals included recipients of either vaccine. Although all women and children were randomized, only a subset participated in the trial. Of the total eligible population (N=121,982), 49,300 women and children received two or more doses of vaccine. The 121,982 individuals lived in 6,415 baris, i.e., clustered patrilineal households, all of which were included in the analysis.

Because this was an individually randomized study, neighborhoods (clusters) were defined from geo-referenced data on the baris. Clusters were formed by a single-linkage agglomerative clustering method. The vaccination coverage varied by cluster, mostly between 20% and 80% coverage. The total number of groups was set to 700. The analysis was based on the difference of the IPW-adjusted average outcomes in the relevant groups. Direct effects were estimated at varying levels of coverage. The indirect, total, and overall effects compared incidence between two different levels of coverage. The direct effect estimates generally decreased with increasing coverage. At the higher coverage, the vaccine did not seem to have a significant direct effect. This illustrates the limitations of analyses that consider only direct effects when interference is present. The indirect and overall effects increased with increasing difference in levels of coverage. The total effects were fairly invariant to the difference in levels of coverage, possibly suggesting that being vaccinated was more important than the indirect effect. Chakladar et al. (2020) considered an extension of the Tchetgen Tchetgen & VanderWeele (2012) IPW estimator to the setting where the outcome is subject to right censoring using inverse probability censoring weights. Censoring weights were estimated using proportional hazards frailty models.

### 6.3. One-Stage Randomized Trials with Control Vaccine

Another vaccine trial design is a cluster-randomized trial with a control vaccine in the clusters randomized not to receive the vaccine of interest. Kilpatrick et al. (2020) consider estimands and estimators for indirect, total, and overall effects in trials where clusters of individuals are randomized to vaccine or control. They examine the scenario where individuals self-select whether to participate in the trial and the outcome of interest is measured on all individuals in each cluster. They show that due to randomization and blinding because of the control vaccine, the indirect, total, and overall effects are estimable from the observed data in such trials. However, the direct effect is in general not estimable without further assumptions.

Diallo et al. (2019) conducted a double-blind, cluster-randomized trial in Senegal. Villages were randomly allocated (1:1) for high-coverage vaccination of children aged 6 months through 10 years with either the 2008–2009 Northern Hemisphere, trivalent influenza vaccine (IIV3) or an inactivated polio vaccine (IPV). All village residents, vaccinated and unvaccinated, were monitored for signs and symptoms of influenza illness using weekly home visits and surveillance in designated clinics. The primary outcome was all laboratory-confirmed symptomatic influenza. Between May 23 and July 11, 2009, 20 villages were randomized, and 66.5% of age-eligible children were enrolled (3,918 in IIV3 villages and 3,848 in IPV villages). Follow-up continued until May 28, 2010. Among vaccinees, the total effectiveness against illness caused by the seasonal influenza virus (presumed to all be drifted A/H3 N2, based on antigenic characterization data) circulating at high rates among children was 43.6% (95% CI, 18.6%−60.9%). The indirect effectiveness against seasonal A/H3 N2 was 15.4% (95% CI, −22.0%−41.3%). The influenza A/H1 N1 pandemic occurred that year, and the vaccine was a mismatch. The total effectiveness against illness caused by the pandemic influenza virus (A/H1N1pdm09) was −52.1% (95% CI, −177.2%−16.6%).

Niang et al. (2021) report results of years 2 and 3 of the same trial, where years 2 and 3 were analyzed as consecutive cluster-randomized controlled trials of IIV3. Again the 20 villages received annual vaccination with IIV3 or IPV of age-eligible residents (6 months to 10 years). The primary outcome was total vaccine effectiveness against laboratory-confirmed influenza illness among age-eligible children. They vaccinated 74% of 12,408 age-eligible children in year 2 (June 2010-April 2011) and 74% of 11,988 age-eligible children in year 3 (April 2011-December 2011) with study vaccines. Annual cumulative incidence was 4.7 (year 2) and 4.2 (year 3) per 100 child vaccinees of IPV villages. In year 2, IIV3 matched circulating influenza strains. The total effectiveness was 52.8% (95% CI, 32.3%−67.0%), and the population effectiveness was 36.0% (95% CI, 10.2%−54.4%) against laboratory-confirmed illness caused by any influenza strain. The indirect effectiveness against A/H3 N2 was 56.4% (95% CI, 39.0%−68.9%). In year 3,74% of influenza detections were vaccine-mismatched to circulating B/Yamagata and 24% were vaccine-matched to circulating A/H3N2. The year 3 total effectiveness was −14.5% (95% CI, −81.2%−27.6%). Vaccine effectiveness varied by type/subtype of influenza in both years.

### 6.4. Estimating Indirect, Total, and Overall Effects from Routinely Collected Data

It is possible to estimate indirect, total, and overall effects from routinely collected data or large databases. The analysis can be adjusted for measured potential confounders. Rane & Halloran (2021) conducted a retrospective cohort study of children born between January 1, 2008, and December 31, 2017, in King County, Washington, who were enrolled in the Washington State Immunization Information System. Diphtheria, tetanus toxoid, and acellular pertussis (DTaP) vaccination data from the Washington State Immunization Information System were linked with pertussis case data from Public Health Seattle and King County. Census-tract-level vaccination coverage was estimated as proportion of age-appropriately vaccinated children residing in it. Direct vaccine effectiveness was estimated by comparing pertussis risk in fully vaccinated and undervaccinated children. Population-level vaccine effectiveness measures were estimated by comparing pertussis risk in census tracts in the highest quartile for vaccination coverage with that in the lowest quartile. Estimated direct protective vaccine effectiveness was 76% (95% CI, 63%−84%) in low-vaccination-coverage clusters, and it decreased to 47% (95% CI, 13%−68%) in high-coverage clusters, after adjustment for potential confounders. The estimated indirect vaccine effectiveness was 45.0% (95% CI, 1%−70%), the total effectiveness was 93.9% (95% CI, 91%−96%), and the overall effectiveness was 42.2% (95% CI, 19%−60%). Their findings suggest that DTaP vaccination provided direct as well as indirect protection in the highly immunized King County, Washington. Routine DTaP vaccination programs may have the potential to provide protection not only for vaccinated individuals but also for the undervaccinated individuals living in the same area.

## 7. IMMUNE CORRELATES AND SIEVE ANALYSIS

Descriptions of the current methods for correlates analysis and sieve analysis are beyond the scope of this review, but they are often built into the design of vaccine trials. We present them briefly for completeness and present a couple of recent examples.

### 7.1. Immune Correlates

A goal of vaccine research is to identify a vaccine-induced immune response that predicts protection from infection and/or disease. Identifying such immune responses could eventually allow licensure of vaccine candidates without additional Phase III trials. It is also important for bridging from study populations to other populations, such as different age groups or geographic locations. Gilbert & Hudgens (2008), among others, proposed a framework for assessing correlates of protection in vaccine trials that delineates different levels of confidence in immunological markers. The framework is based on the methods of Prentice (1989) and Frangakis & Rubin (2002). A correlate of risk (CoR) is an immunological measurement that predicts a clinical endpoint in a particular population. A correlate, or surrogate, of protection (CoP) is a CoR that also predicts the level of protective efficacy of a vaccine. Identifying CoRs and CoPs is often a secondary objective of vaccine trials. Plotkin & Gilbert (2012) clarified some of the terminology.

Much interest centered around the correlates analysis of the COVID-19 vaccine trials. Gilbert et al. (2022) presented an immune correlates analysis of the mRNA-1273 COVID-19 vaccine trial. In the Phase III trial, vaccine recipients were assessed for neutralizing and binding antibodies as correlates of risk for COVID-19 disease and as correlates of protection. All markers were inversely correlated with COVID-19 risk and directly associated with vaccine efficacy. Fong et al. (2023) presented a similar analysis of the NVX-CoV-2373 Phase III trial, a different vaccine. They also found that all markers were inversely correlated with COVID-19 risk and directly associated with vaccine efficacy. Fong et al. (2023) suggested that their results support potential cross-vaccine platform applications of these markers for guiding decisions about vaccine approval and use.

Causal mediation analysis provides a different framework to quantify the role of various immune mediators of protection. Cowling et al. (2019) and Lim et al. (2024) used causal mediation analysis to estimate the proportion of the total effect of influenza vaccination that was meditated by higher hemagglutination inhibition antibody titers.

### 7.2. Sieve Analysis

A key component in the evaluation of efficacy of a vaccine to protect against disease caused by an antigenically diverse genetic pathogen in a vaccine trial is assessing how vaccine-induced protection depends on genotype and phenotype variations of the exposing pathogen. The evaluation is done by comparing pathogen isolates between infected vaccinated participants and infected unvaccinated participants. This is called sieve analysis (Gilbert et al. 2001). Recent examples of sieve analysis for different pathogens and vaccines include a malaria vaccine (Neafsey et al. 2015) and a dengue vaccine (Juraska et al. 2018). Bai et al. (2024) use methods related to sieve analysis to design better HIV vaccine candidates.

## SUMMARY POINTS

1. Direct protective effects of vaccines are just one aspect of vaccine protection.
2. Several recent observational study designs allow evaluation of various vaccine effects.
3. Emerging infectious diseases have provided the impetus for novel design of vaccine trials.
4. Evaluating effects of vaccines on transmission is important both to understand the effects and to provide estimates for population models.
5. Vaccination in populations can have indirect effects on the unvaccinated, making vaccination more cost-effective.
6. The appropriate study design needs to be chosen to evaluate the effect of interest to the scientific question.

## FUTURE ISSUES

1. Real-world evidence of vaccine effectiveness in settings beyond clinical trials will be important.
2. Observational trial designs will be more widely used to evaluate vaccines in the field.
3. Evaluating indirect effects of vaccination will be important to understand vaccine costeffectiveness.
4. Platform vaccine trials will be useful for evaluating vaccines for emerging infectious diseases.
5. Immune correlates analyses will become more widespread for licensing new vaccines.
